# Acute Partial Brown-Séquard Syndrome Secondary to Intraforaminal Disc Prolapse and Spinal Cord Infarction

**DOI:** 10.1155/2019/7987038

**Published:** 2019-09-25

**Authors:** Athanasios Astreinidis, Stephanos Finitsis, Xanthippi Mavropoulou, Elisavet Psoma, Panagiotis Prassopoulos

**Affiliations:** ^1^Department of Endovascular Surgery, AHEPA Hospital, Aristotle University of Thessaloniki, Panepistimioupoli, 54124 Thessaloniki, Greece; ^2^Department of Radiology and Medical Imaging, AHEPA University Hospital, Panepistimioupoli, 54124 Thessaloniki, Greece

## Abstract

We report the case of a 45-year-old female who presented with acute left abdominal pain and subsequently developed a left partial Brown-Séquard syndrome. Spinal fluid, inflammatory and prothrombotic tests were unremarkable. Magnetic resonance showed a left intraforaminal disc prolapse at the T9–T10 level and a hyperintense lesion on T2-weighted images in the left postero-lateral cord at the T8–T9 level with restricted diffusion on DWI imaging. A diagnosis of spinal cord infarction due to compromise of the left T8 thoracic radicular artery was made. The patient was managed conservatively and at the 3 months follow-up, she was ambulant and able to walk small distances without a walker.

## 1. Introduction

A 45-year-old female with no prior medical history and a body mass index of 25 abruptly developed left abdominal pain while seated. She subsequently developed left lower limb weakness and contralateral paraesthesia.

Admitted 24 hours post ictus, hyperreflexia and severe paresis was found involving all muscle groups of the left lower limb with minimally preserved extension of the thigh (grade 2/5) and foot (grade 2/5). Left kinaesthesia was slightly reduced. Profound sensory loss was noted below the T9–T10 level on the left. Pain sensation was diminished in the right lower limb, while pyramidal function was normal. Proprioception and vibration were symmetrical.

Blood tests including levels of CRP, RF, immunoglubolin, protein electrophoresis and immunofixation, prethrombotic tests including Factor V-Leiden, prothrombin mutations, levels of AT3, quantity of protein C, lupus anticoagulation and homocysteine levels, as well as CSF examination including biochemical examination, levels of glucose, protein ANA, AMA, ANCA, ASMA autoantibodies and cultures were negative.

MRI of the head and cervical spinal cord, cardiac echo, and abdominal echo were unremarkable.

**Figure 1:**
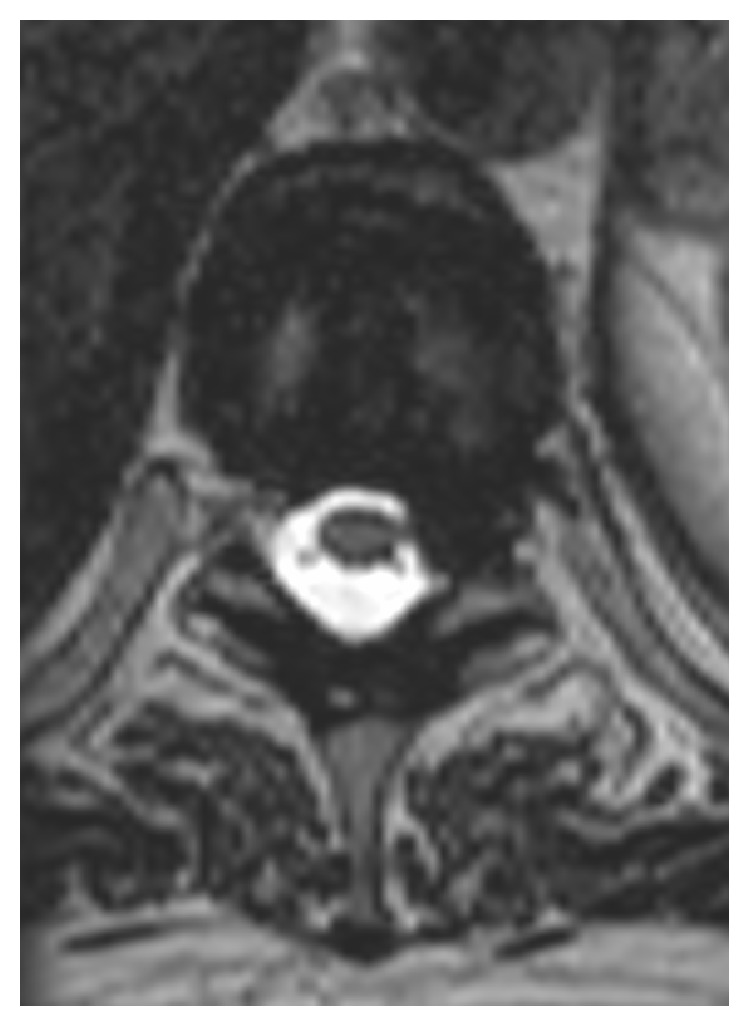


**Figure 2:**
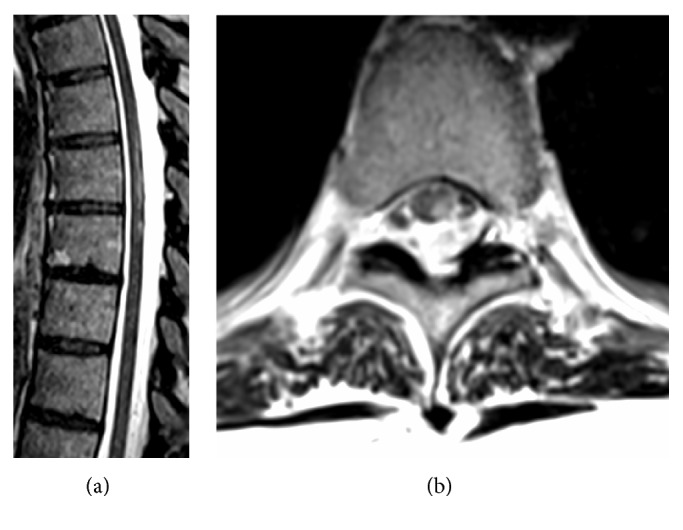


**Figure 3:**
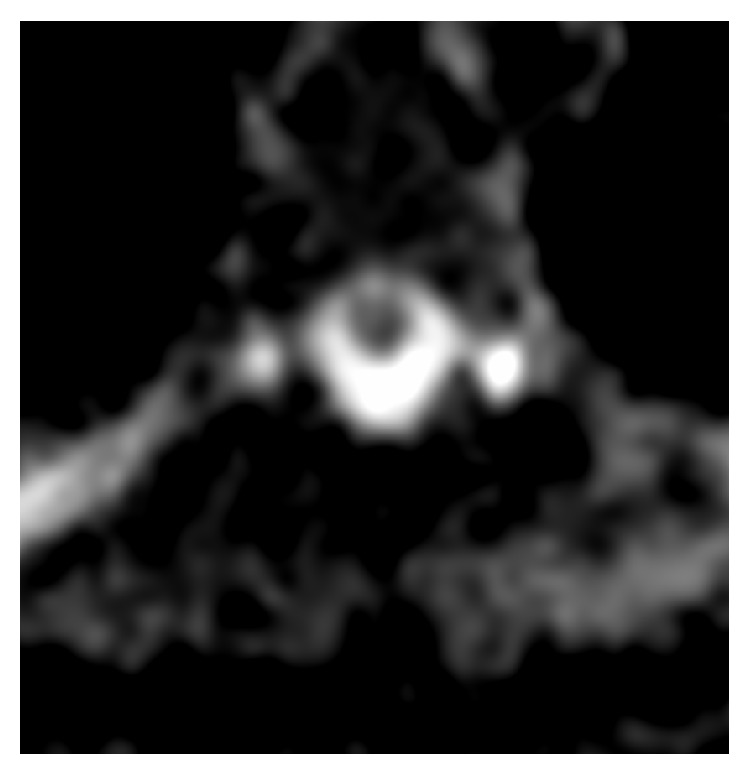


The signs and symptoms are consistent with a partial BSS on the left at the level of T9–T10. Thoracolumbar MRI demonstrates a left intraforaminal disc protrusion at T9–T10 with no significant compression of the spinal cord (Figure 1). On T2 weighted sequences, there is a high intensity lesion in the left postero-lateral spinal cord extending from T8 to T9 (Figure 2). On DWI the lesion has a reduced ADC value (Figure 3).

The patient was managed conservatively with intravenous administration of glucocorticoids (methylprednisolone 1 gr/day for 3 days, tapered for another 3 days), mannitol (400 mg/day for 4 days), and subcutaneous enoxaparin 75 mg/12 h for 1 week. Three months later, the patient was ambulant and could walk small distances without a walker. No further clinical improvement was observed after 6 months and 12 months. Control MRI showed no residual lesion on T2-weighted sequences without restriction on DWI images, while the disc prolapse had resolved completely.

The protruding thoracic disk produced an acute partial Brown-Séquard syndrome most probably either by direct compression or by disk fragment embolization of the left T8 radicular artery. This is the third case of partial Brown-Séquard syndrome secondary to disc protrusion reported in the literature. In the first case, the artery of Adamkiewicz was intraoperatively demonstrated to be compressed, while in the second case FCE was probably the aetiology of ischemia because of coexisting ischemic changes in the neighbouring vertebral body [5]. Our case also highlights the use of DWI in the acute setting of SCI to establish the ischemic nature of a spinal cord lesion when adjacent vertebral bone marrow ischemic changes are lacking.

## 2. Discussion

SCI is rare among younger patients and its aetiology often remains unexplained in more than 50% of cases. Differential diagnoses which have to be considered are spinal contusion, spinal hypoperfusion, acute transverse myelitis, and cord compression subsequent to medial disc herniation or tumour growth [1].

The typical BSS comprises a unilateral spinal lesion, resulting in ipsilateral loss of motor function, interruption of the ascending fibres in the posterior white column resulting in ipsilateral loss of tactile discrimination, and of vibratory and positional sensation. Additionally, BSS is characterized by spinothalamic tract dysfunction resulting in contralateral loss of pain and temperature sensation. The syndrome is most frequently observed in association with traumatic injuries to the spinal cord and extramedullary spinal cord tumours [2].

The entire spinal column and its structures receives their blood supply from 31 pairs of segmental arteries derived mainly from vertebral arteries or the aorta. The intraspinal branches of the segmental arteries which enter the spinal canal through the intervertebral foramina are called radicular arteries. They accompany the exiting nerves and veins and have an ascending course from caudal to cranial. Each radicular artery supplies the nearby bone and soft tissues, and divides into an anterior and posterior branch. Concerning the spinal cord, the anterior two-thirds and the centre are supplied by the ASA that runs superficially to the anterior median fissure and gives of sulcocomissural arteries that penetrate the fissure. The posterior third of the spinal cord is supplied by the paired PSAs. Both ASA and PSAs extend from the medulla to the conus medullaris [3]. The ASA is supplied by 2 radicular arteries through their anterior branch, while the PSA is formed by 10–23 radicular arteries through their posterior branch. A radicular branch suppling the spinal cord is called a radiculomedullary artery. The artery of Adamkiewicz is the largest anterior radiculomedullary artery and usually supplies a large portion of the thoracolumbar spinal cord. Besides supplying the ASA and PSAs, the radiculomedullary branches supply the vasa corona, a fine arterial network that gives perforators to the circumference of the cord.

The diagnosis of acute spinal cord ischemia is often clinical based on an abrupt presentation of a neurological deficit and back pain in 70% of cases. The most common artery involved is the ASA, resulting in an ASA syndrome. The PSA syndrome is relatively infrequent, while total transverse spinal cord ischemia involving both ASA and PSA has rarely been described. The rare cases of incomplete Brown-Séquard syndrome are thought to be secondary to involvement of sulcocomissural arteries.

Acute SCI may manifest on MRI as an intramedullary hyperintensity on T2-weighted images with cord enlargement. However, these signs are visible in approximately 45% of patients. When the occlusion involves vessels supplying the vertebrae, vertebral body infarction adjacent to a cord abnormality may be found on MRI in 4%–35% of cases and is a confirmatory sign of ischemia. Vertebral body infarctions are usually seen in the thoracolumbar region and are often associated with aortic disease. The bone marrow abnormality of the vertebra tends to appear earlier and be more exaggerated compared to the cord. DWI is more sensitive than T2 weighted images and may show ischemic lesions earlier than conventional MRI. When an infarction of the vertebral body is not observed, as in our case, DWI may be used to confirm the diagnosis of cord ischemia. Another important role of MRI is to rule out compressive myelopathies, vascular malformations, infective myelitis, demyelinating disorders, and tumours.

Atherosclerosis, aortic dissection (3%–5% risk), and aortic surgery (1%–10% risk) are the most common causes of acute SCI. Other rare causes include cardiac embolism, decompression sickness, coagulopathy, spinal arteriovenous malformations, systemic hypotension, epidural anaesthesia, trauma, and vasculitis. However, in 50% of patients, the aetiology remains unclear. Rarely, SCI may be due to mechanical compromise of a radicular artery following minor trauma. It is postulated that a sudden change of intervertebral disc pressure, especially in a degenerative disc may lead to rupture and penetration of cartilaginous material into neighbouring spinal vessels. FCE is thought to be a rarely recognized but important cause of SCI [4]. However, FCE lacks definitive diagnostic criteria.

Usually there is acute severe pain and the neurological deficit appears 15 min to 48 hours or later. MRI should be compatible with SCI, the cerebrospinal fluid is normal and inflammatory or autoimmune causes of myelopathy should be excluded. Different treatments options like intravenous steroids, plasma exchange, iv heparin, and emergent decompressive surgery have been tried without much success. The functional outcome varies from moderate to severe disability or even death if the cervical region is involved.

In the present case, control MRI at 3 months failed to show persisting T2 signal changes though the patient had a clear neurological deficit, while the disk prolapse had resolved completely. We suspect that the remaining spinal cord lesion was beyond the spatial resolution of the MRI equipment. The complete resolution of the disk prolapse in such a short time span attests to the dynamic nature of the lesion.

## Abbreviations

SCI: Spinal cord ischemia
FCE: Fragment cartilage embolization
BSS: Brown-Séquard syndrome
ASA: Anterior spinal artery
PSA: Posterior spinal artery.
